# A framework for sample size calculations in longitudinal surveys to measure net and gross changes

**DOI:** 10.1371/journal.pone.0291449

**Published:** 2023-09-21

**Authors:** Mahmoud Elkasabi, Z. Tuba Suzer-Gurtekin, Yuxuan Chen

**Affiliations:** 1 Center of Official Statistics, RTI International, District of Columbia, United States of America; 2 Institute for Social Research, University of Michigan, Ann Arbor, Michigan, United States of America; National Institute of Technology Silchar, India, INDIA

## Abstract

In longitudinal surveys, repeated measurements are collected from the same sample units over time to measure *gross change* (i.e., change at the level of individual sample members). Longitudinal samples are sometimes supplemented by fresh sample to measure *net change* (i.e., change at the aggregate level). That is, in each measurement wave, while one part of the sample is newly recruited (fresh), another part overlaps with previously interviewed sample (repeated interviews). Many aspects of survey design of longitudinal surveys have been studied extensively, such as definition of target population, sample design, survey weighting, intervals between interviews, nonresponse, and panel attrition. Although the impact of the overlap between samples on the statistical power has been studied, sample size determination lacks a formulation that takes account of these factors in longitudinal surveys that aim to measure net and gross changes simultaneously. In this study, we propose a framework for sample size calculation to measure net and gross changes in estimated means or proportions concurrently in longitudinal surveys. We present a framework to compute panel and fresh sample sizes for varying levels of net and gross change. Finally, we illustrate the framework using *nchange*, an R package we developed to execute the algorithm of the proposed framework. The framework and the R package will support researchers to determine sample sizes targeting specific power of analysis with respect to measuring net and gross changes in rotating- or split-panel surveys.

## 1. Background

Longitudinal surveys collect measurements from the same sample units over time for a prespecified number of survey waves. The term *longitudinal surveys* is widely used to describe different survey designs, including rotating- and split-panel designs. Both designs collect measurements from a combination of fresh and repeated interviews at every data collection wave. That is, in addition to interviewing a sample of entities over time at multiple time points, a new sample of entities is interviewed as well. In rotating-panel designs, such as the University of Michigan Surveys of Consumers (SoC) [1], a proportion of sample units are replaced with new sample units in each wave, and each sample unit remains in the sample for a fixed number of survey waves. In split-panel designs, a combination of cross-sectional and panel samples is used at each survey wave [2–6].

Duncan and Kalton [7] outlined the analytical properties of rotating- and split-panel survey designs along with those of other designs. For example, rotating- and split-panel designs allow estimating net change (i.e., change at the aggregate level) in addition to gross change (i.e., change at the element level between two time points simultaneously). At the same time, repeated measurements allow for research on the dynamics of causation and relationships ([8], p. 470). For example, Johnson, Pence, and Vine [9] used panel data from the SoC to investigate the impact of consumers’ assessments of vehicle financing conditions on new car purchases. Furthermore, with such overlapped repeated measurements designs, causality and growth patterns can be modeled using hazard, survival, and latent growth models [6]. Finally, apart from measuring changes, longitudinal surveys allow for cumulating data across time intervals, for example monthly or annual surveys can be combined yielding increased sample sizes for subgroups of interest [7].

In this research note, we focus on (1) net change in estimated means or proportions (difference between estimates from two samples of two survey waves), and (2) gross change in estimated means or proportions (difference between estimates only from the overlapped samples of two survey waves). Although the net change estimates provide information on stability at the aggregate level, a number of studies showed that such information is not enough especially for public policy decision makers who need information on the gross change as well [10]. For example, although the U.S. Census Bureau reported little net change in poverty rate in the 1970s, results from the Panel Study of Income Dynamics indicate a gross decline in poverty rate around the same period (i.e., the same families did not stay poor over the same period) [11–15].

Sample size for probability samples is determined using precision analysis or power analysis [16–18]. In precision analysis, sample size is determined to estimate an unknown parameter, such as an estimated proportion, mean, odds ratio, or relative risk, with a prespecified precision at a fixed significance level. Prespecified values of standard error, margin of error, or coefficient of variation are typically used as indicators for precision. In power analysis, sample size is determined to achieve a desired power for detecting changes in estimated parameters at a fixed significance level. These changes might be differences in estimated means, proportions, odds ratios, or relative risks. The estimated parameters are typically measured from two independent samples—control and treatment groups—based on an experimental design or baseline and intervention surveys. Our colleagues [17] addressed a special case where sample size is determined to achieve a desired power for detecting changes between estimates that are measured from two overlapped samples. Because the portion of the changes across waves is measured based on the same sample units, the required sample size for longitudinal surveys is lower than that of repeated cross-sectional surveys. Although the literature focuses on showing the change in precision of net change estimates related to the intraclass correlations of repeated measurements [7], these general approaches to design with overlapping units cite objectives as measures of level and net change but do not incorporate the gross change precision determination.

According to lynn [6], “There are some aspects of survey design that are unique to longitudinal surveys, or are substantially different in nature to cross-sectional surveys. Standard survey methods textbooks provide little or no guidance on how to make design decisions regarding these aspects. Yet these aspects warrant careful consideration as design decisions can have weighty implications for data quality and for analysis possibilities” (p. 11). Lynn [6] provides a good summary for those aspects, such as definition of target population, sample design, weighting, intervals between waves, nonresponse, and panel attrition. Unfortunately, although sample size calculation is a key element in designing sample surveys, this aspect needs more attention with respect to longitudinal surveys, especially when several survey objectives need to be addressed.

This research note addresses a common scenario where measuring net and gross change are two key objectives of a longitudinal survey and can follow either a rotating- or split-panel design. In this research note, we outline a framework to compute sample sizes that focuses on the dual objectives of net and gross change estimates. We also introduce *nchange*, an R package we developed to execute the algorithm of the proposed framework. In the next section, we review the calculation of sample size to detect net and gross change. We then introduce a framework for calculating the sample size for detecting both changes. Finally, we present an illustration for the execution of the framework using the R package.

## 2. Sample size calculation to detect net and gross changes

In this section, we review the sample size calculation for detecting net and gross changes between two time points, *t* and *t+1*, using samples from a split or a rotating panel design. Let *s*_*t*_ and *s*_*t+1*_ denote two samples of size *n*_*t*_ and *n*_*t+1*_, where *s*_*10*_ denotes a sample subset of *n*_*10*_ units with data collected only at time *t*, *s*_*11*_ denote a sample subset of *n*_*11*_ units with data collected at times *t* and *t+1*, and *s*_*01*_ denote a sample subset of *n*_*01*_ units with data collected at time *t+1* only. This structure implies that *s*_*t*_ = *s*_10_ ∪ *s*_11_ and *s*_*t*+1_ = *s*_01_ ∪ *s*_11_.

### 2.1. Net change

When both *s*_*t*_ and *s*_*t+1*_ are simple random samples and *y*_*i*_ and *x*_*i*_ represent the attribute measured at *t* and *t+1* for the *i*^*th*^ unit, the net change in means can be written as

$$\hat{\bar{\delta }}=\frac{1}{n_{t}}\sum_{i\in s_{10}}y_{i}-\frac{1}{n_{t+1}}\sum_{i\in s_{01}}x_{i}+\sum_{i\in s_{11}}\left(\frac{y_{i}}{n_{t}}-\frac{x_{i}}{n_{t+1}}\right)$$ (1)

and the variance of $\hat{\bar{\delta }}$ is

$$V\left(\hat{\bar{\delta }}\right)=\frac{\sigma_{x}^{2}}{n_{t}}+\frac{\sigma_{y}^{2}}{n_{t+1}}-2\sigma_{xy}\frac{n_{11}}{n_{t}n_{t+1}}$$ (2)

where $\sigma_{x}^{2}$ and $\sigma_{y}^{2}$ denote population variance of *X* and *Y*, and *σ*_*xy*_ denotes the population covariance of *X* and *Y*. The required sample size to detect a net change *δ* can be calculated as

$$n_{t}=\frac{1}{\delta^{2}}\left[\sigma_{x}^{2}+r\sigma_{y}^{2}-2\gamma r\rho \sigma_{x}\sigma_{y}\right]{\left(Z_{1-\alpha /2}-Z_{\beta }\right)}^{2}$$ (3)

where *r* = *n*_*t*_/*n*_*t*+1_, *γ* = *n*_11_/*n*_*t*_, *ρ* = *σ*_*xy*_/*σ*_*x*_*σ*_*y*_, *Z*_1−*α*/2_ is the 100(1- *α*/2)^th^ percentile normal distribution that corresponds to the desired level of significance (*α*) for 2-sided test *H*_*o*_: *δ* = *d* versus *H*_*A*_: *δ* ≠ *d*, and *Z*_*β*_ corresponds to the desired power of test (1 − *β*) for the net change. Eq (3) is the two-sided test version of equation (4.13) in [17].

When the study variable is a binary outcome with 0/1 values, the difference in means reduces to a difference in proportions *P*_*t*_ − *P*_*t*+1_, and the required sample size to detect a net change *δ* can be calculated as

$$n_{t}=\frac{P_{t}\left(1-P_{t}\right)+rP_{t+1}\left(1-P_{t+1}\right)-2\gamma r\left(P_{XY}-P_{t}P_{t+1}\right)}{\delta^{2}}{\left(Z_{1-\alpha /2}-Z_{\beta }\right)}^{2}$$ (4)

where *P*_*XY*_ denotes the proportion of units in *s*_*11*_ with the same study characteristic in *t* and *t+1*. See the proof of (4) in Appendix A in S1 Appendix.

As is clear from Eqs (3) and (4), the more correlation of *ρ* or overlap of *γ*, the less sample size *n*_*t*_ is required to detect the same net change *δ*. This implies that increasing the panel share *γ* can simultaneously increase statistical power for detecting changes while reducing the required sample size (i.e., the costs of conducting the survey). Previous research focused on incorporating this characteristic in computing precision for net change using rotating panel or split panel designs [6, 7].

### 2.2. Gross change

Unlike with net change, units that existed at only one of the two time points, *s*_*10*_ and *s*_*01*_, do not contribute to the calculation of gross change. Only units that are measured in the two time points contribute to gross change. Gross change Δ is defined based on *s*_*11*_ only as below

$$\hat{\bar{\Delta }}=\frac{1}{n_{11}}\sum_{i\in s_{11}}\left(y_{i}-x_{i}\right)$$ (5)

where $\sigma_{x}^{2}=\sigma_{y}^{2}\equiv \sigma_{o}^{2}$, the sample size of *n*_*11*_ can be calculated as

$$n_{11}=\frac{2\sigma_{o}^{2}}{\Delta^{2}}\left(1-\rho \right){\left({\overset{´}{Z}}_{1-\overset{´}{\alpha }/2}-\;{\overset{´}{Z}}_{\overset{´}{\beta }}\right)}^{2}$$ (6)

where ${\overset{´}{Z}}_{1-\overset{´}{\alpha }/2}$ corresponds to the desired level of significance ($\overset{´}{\alpha }$) for the gross change, and ${\overset{´}{Z}}_{\overset{´}{\beta }}$ corresponds to the desired power of test $\left(1-\overset{´}{\beta }\right)$ for the gross change.

When the study variable is a binary outcome with 0/1 values, gross change can be measured as a difference between proportions, ${\overset{´}{P}}_{t}$ and ${\overset{´}{P}}_{t+1}$, calculated based on *n*_*11*_ as $\frac{1}{n_{11}}\sum_{i\in s_{11}}\left(y_{i}-x_{i}\right)$, and the required sample size to detect a gross change *Δ* can be calculated as

$$n_{11}=\frac{{\overset{´}{P}}_{t}\left(1-{\overset{´}{P}}_{t}\right)+{\overset{´}{P}}_{t+1}\left(1-{\overset{´}{P}}_{t+1}\right)-2\left({\overset{´}{P}}_{XY}-{\overset{´}{P}}_{t}{\overset{´}{P}}_{t+1}\right)}{\Delta^{2}}{\left({\overset{´}{Z}}_{1-\overset{´}{\alpha }/2}-\;{\overset{´}{Z}}_{\overset{´}{\beta }}\right)}^{2}$$ (7)

Both Eqs (6) and (7) can be easily derived from Eqs (3) and (4), respectively, as *r* = 1 and *γ* = 1.

Depending on available data, we need to think about different scenarios to compute sample sizes to measure net and gross changes concurrently. The amount of available information imposes a different problem parameterization as we advance from *t* to *t+1* in data collection. For example, before time *t*, the problem is to find *n*_*t*_, *n*_*11*_ and *n*_*t+1*_ of samples *s*_*t*_ and *s*_*t+1*_, whereas between times *t* and *t+1*, the problem is to solve for *n*_*11*_ and *n*_*t+1*_ of sample *s*_*t+1*_ given the available *n*_*t*_ from sample *s*_*t*_. In the later scenario, when *n*_*t*_ is known, the required sample size *n*_*t+1*_ to detect a net change *δ* in a continuous variable can be calculated as

$$n_{t+1}=\frac{\left[n_{t}\sigma_{y}^{2}-2n_{11}\rho \sigma_{x}\sigma_{y}\right]{\left(Z_{1-\alpha /2}-Z_{\beta }\right)}^{2}}{n_{t}\delta^{2}-\sigma_{x}^{2}{\left(Z_{1-\alpha /2}-Z_{\beta }\right)}^{2}}$$ (8)

where

$$n_{11}=\frac{2\sigma_{o}^{2}}{\Delta^{2}}\left(1-\rho \right){\left({\overset{´}{Z}}_{1-\overset{´}{\alpha }/2}-\;{\overset{´}{Z}}_{\overset{´}{\beta }}\right)}^{2}$$ (9)

See Appendix A in S1 Appendix for a proof of Eq (8). Eq (9) is based on Eq (6). To detect a net change *δ* in proportions of binary variable, the following equations can be used:

$$n_{t+1}=\frac{\left[n_{t}P_{t+1}\left(1-P_{t+1}\right)-2n_{11}\left(P_{XY}-P_{t}P_{t+1}\right)\right]{\left(Z_{1-\alpha /2}-Z_{\beta }\right)}^{2}}{n_{t}\delta^{2}-P_{t}\left(1-P_{t}\right){\left(Z_{1-\alpha /2}-Z_{\beta }\right)}^{2}}$$ (10)

where

$$n_{11}=\frac{{\overset{´}{P}}_{t}\left(1-{\overset{´}{P}}_{t}\right)+{\overset{´}{P}}_{t+1}\left(1-{\overset{´}{P}}_{t+1}\right)-2\left({\overset{´}{P}}_{XY}-{\overset{´}{P}}_{t}{\overset{´}{P}}_{t+1}\right)}{\Delta^{2}}{\left({\overset{´}{Z}}_{1-\overset{´}{\alpha }/2}-\;{\overset{´}{Z}}_{\overset{´}{\beta }}\right)}^{2}$$ (11)

See Appendix A in S1 Appendix for a proof of Eq (10). Eq (11) is based on Eq (7). Unfortunately, finding similar closed form formulas when *n*_*t*_, *n*_*11*_, and *n*_*t+1*_ are unknown is not easy (see Appendix B in S1 Appendix for details). Therefore, as illustrated in the next section, we propose a sequential algorithm to simulate the achieved powers using different scenarios of *n*_*t*_, *n*_*11*_, and *n*_*t+1*_ and choose the final sample size accordingly.

## 3. Proposed framework for sample size calculations under dual goal

**Fig 1:**
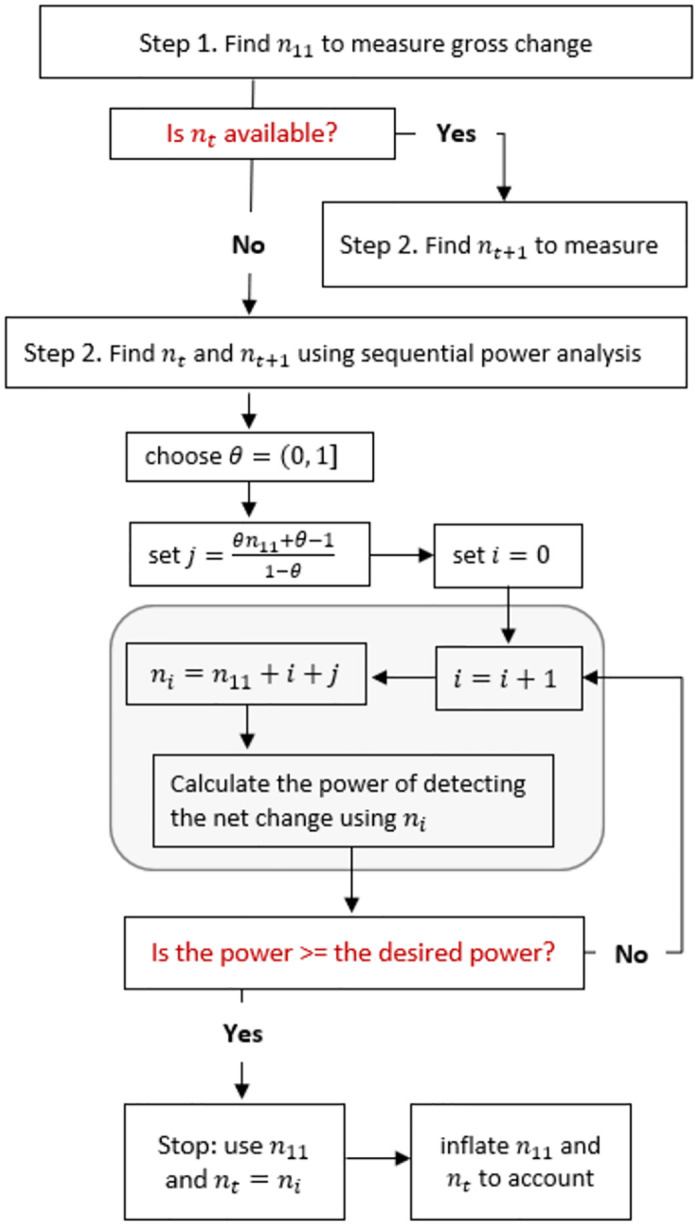
Flowchart of the proposed algorithm for sample size calculation.

In this section, we propose a framework to find *n*_*t*_ and *n*_11_ that are enough to (1) achieve the desired power (1 − *β*) for detecting net change *δ* at a significance level *α*, and (2) achieve the desired power $\left(1-\overset{´}{\beta }\right)$ for detecting gross change Δ at a significance level $\overset{´}{\alpha }$. Because *n*_11_ is one of the determinants of *n*_*t*_, as *n*_*t*_ ∝ 1/*γ*, finding proper values of *n*_*t*_ and *n*_11_ should be done simultaneously. In the proposed framework, first we simulate different data structures and then examine the power of detecting net and gross change simultaneously. To simulate the data structures, we use a two-stage power analysis in which a proper *n*_11_ is determined to achieve the desired power $\left(1-\overset{´}{\beta }\right)$ for detecting gross change Δ at a significance level $\overset{´}{\alpha }$. In the second stage, we sequentially increase *n*_10_ and approximate the achieved power (1 − *β*) for detecting net change *δ* at a significance level *α*. In the proposed framework and illustrations, we assume *r* = 1 as *n*_*t*_ = *n*_*t*+1_; however, the framework can accommodate the other scenario of *r* ≠ 1. As indicated in the flowchart presented in Fig 1, the proposed framework follows the algorithm below:

1. Find the required *n*_*11*_ to measure gross change Δ at a significance level $\overset{´}{\alpha }$ and $\left(1-\overset{´}{\beta }\right)$ power
$$n_{11}=\frac{2\sigma_{o}^{2}}{\Delta^{2}}\left(1-\rho \right){\left({\overset{´}{Z}}_{1-\overset{´}{\alpha }/2}-\;{\overset{´}{Z}}_{\overset{´}{\beta }}\right)}^{2}$$
2. Find the required *n*_*t*_ and *n*_*t+1*_ to measure net change *δ* at a significance level *α* and (1 − *β*) power using a sequential power analysis as follows:
  2.1 Set a lower bound for *n*_*10*_ as
$$j=\frac{\theta n_{11}+\theta -1}{1-\theta }$$
    where 0 ≤ *θ* < 1. When *θ* is set to 0, the lower bound of *n*_*10*_ is set to 0; when *θ* is set to 0.5, the lower bound of *n*_*10*_ is set to equal *n*_*11*_.
  2.2 Set the iteration indicator as *i* = 0.
  2.3 Set the sequential distance of iterations as *i* = *i* + 1.
  2.4 Set an initial value of *n*_*t*_ as *n*_*i*_ = *n*_11_ + *i* + *j*.
  2.5 Approximate the power of detecting the net change *δ* at a significance level *α* using *n*_*t*_ as below
$$Z_{i}=Z_{1-\alpha /2}-\frac{\sqrt{n_{i}\delta^{2}}}{\sqrt{\sigma_{x}^{2}+\sigma_{y}^{2}+2\gamma_{i}\rho \sigma_{x}\sigma_{y}}}$$
    where *n*_*t*+1_ is set to be equal to *n*_*t*_, and *γ*_*i*_ = *n*_11_/*n*_*i*_.
3. Perform a power assessment as follows: If *Z*_*i*_ ≥ *Z*_*β*_, stop and use *n*_11_ and *n*_*t*_ = *n*_*i*_. Otherwise, rerun steps 2.3 to 2.5.
4. Inflate sample sizes to account for panel attrition and nonresponse as below
$${\overset{´}{n}}_{11}=\frac{n_{11}}{RR_{11}}$$
$${\overset{´}{n}}_{10}=\frac{n_{t}-n_{11}}{RR_{10}}$$
  where *RR*_*11*_ and *RR*_*10*_ are completion rates among panel and fresh sampling units respectively.

## 4. Illustration

We designed the *nchange* R package to execute the algorithm of the proposed framework [19]. The main functions “seqmeans” and “seqprop” are designed to execute the proposed algorithms for means and proportions, respectively. The package also accommodates the case of complex sample design by inflating the calculated sample size by an approximated design effect (deff). See Appendix C in S1 Appendix for more details about the function arguments.

### 4.1. Package accessibility

The source code for the *nchange* package is hosted on GitHub at https://github.com/mahmoudelkasabi/nchange, and it can be installed as follows:

install.packages("devtools")

library(devtools)

install_github("mahmoudelkasabi/nchange")

### 4.2. Illustration of sample size for two samples based on differences in means

We would like to find simple random samples, *n*_*t*_, *n*_*11*_, and *n*_*t+1*_, to measure a net increase of *δ* = 3% at a significance level *α* = 0.05 and power (1 − *β*) = 0.80, and a gross increase of Δ = 3% at a significance level $\overset{´}{\alpha }=0.05$ and power $\left(1-\overset{´}{\beta }\right)=0.80$. Assume *r* = 1, $\sigma_{x}^{2}=100,\;\sigma_{y}^{2}=75,\;\sigma_{o}^{2}=200$, and *ρ* = 0.90. This problem can be solved using the *seqmeans* function from *nchange* as below:

library(nchange)

seqmeans(theta = 0.5,rho=0.9,deff=1,S2x=100,S2y=75,

  alt="one.sided",del=3,sig.level=0.05,power = 0.80,S2o=200,

  alt.gross="one.sided",del.gross=3,sig.level.gross=0.05, pow.gross=0.8)

**Fig 2:**
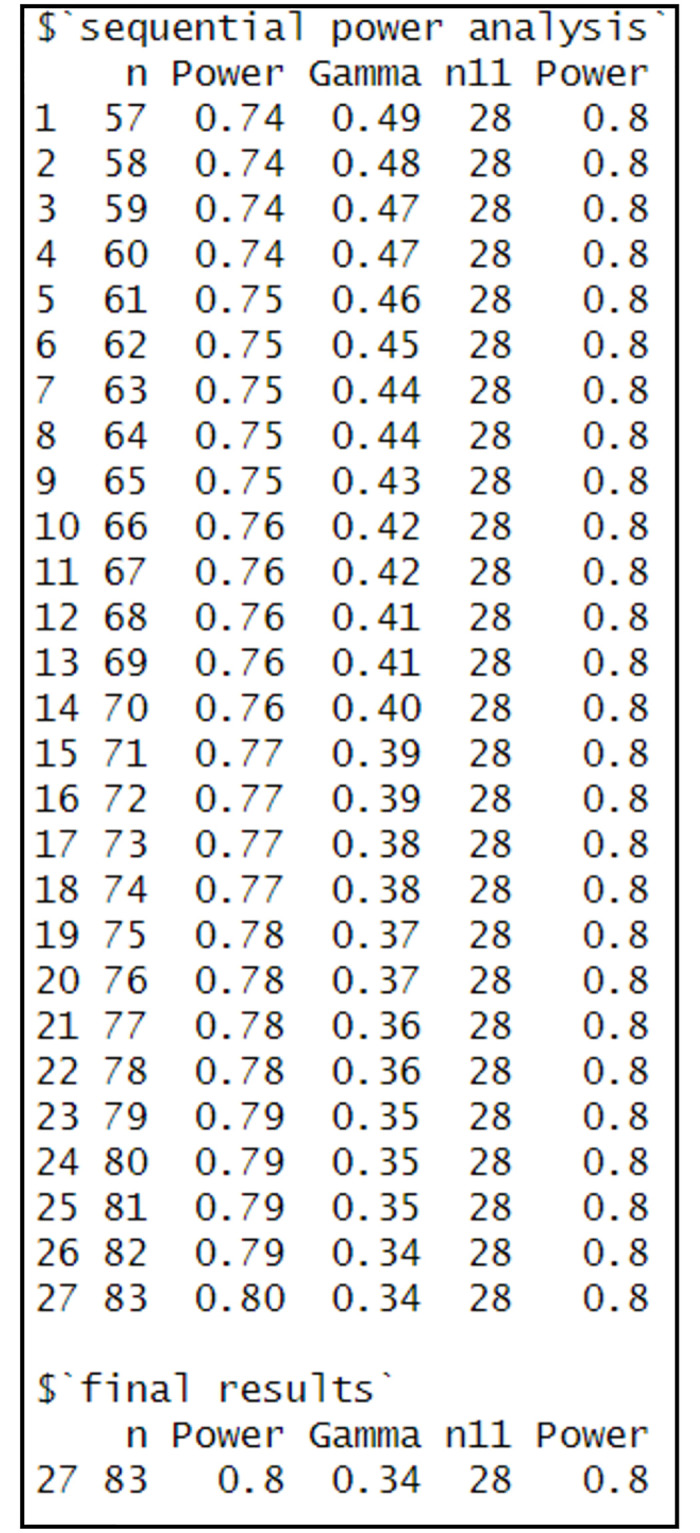
Results of illustration of sample size for two samples based on differences in means.

As indicated from the results in Fig 2, to measure the gross difference, *n*_*11*_ = 28 is required. The equation executed 27 iterations to find a proper *n*_*t*_ to measure the net difference. Based on the final iteration, *n*_*t*_ = 83 and *n*_*11*_ = 28 that implies *γ* = 0.34.

### 4.3. Illustration of sample size for two samples based on differences in proportions

Similarly, we can find *n*_*t*_, *n*_*11*_, and *n*_*t+1*_ to measure changes in proportions using the *seqprop* function. When the net proportions are *P*_*t*_ = 0.50, *P*_*t*+1_ = 0.70, and *P*_*XY*_ = 0.45, and the gross proportions are ${\overset{´}{P}}_{t}=0.50,\;{\overset{´}{P}}_{t+1}=0.80$, and ${\overset{´}{P}}_{XY}=0.45$ with significance and power level similar to the previous example, we can use the following function:

seqprop(theta = 0.5,deff=1,P1=0.5,P2=0.7,PXY=0.45,

  alt="one.sided",sig.level=0.05,power=0.80,

  P1.gross=0.5,P2.gross=0.8,PXY.gross=0.45,

  alt.gross="one.sided",sig.level.gross=0.05,pow.gross = 0.80)

**Fig 3:**
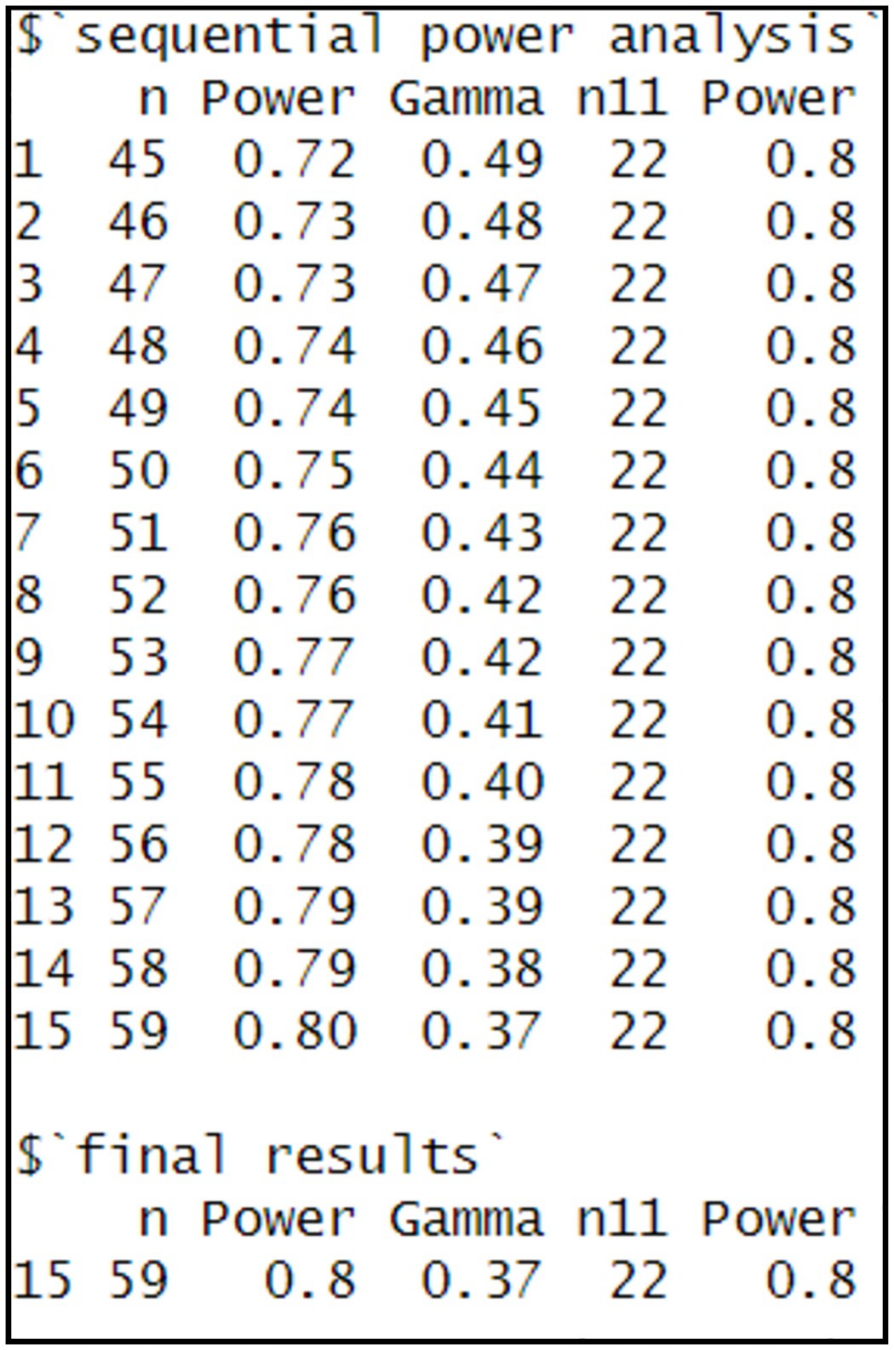
Illustration of sample size for two samples based on differences in proportions.

As indicated from the results in Fig 3, to measure the gross difference, *n*_*11*_ = 22 is required. The equation executed 15 iterations to find a proper *n*_*t*_ to measure the net difference. Based on the final iteration, *n*_*t*_ = 59 and *n*_*11*_ = 22 that implies *γ* = 0.37.

## 5. Conclusion

This article presents a framework to compute overlapping sample sizes in addition to total sample sizes for two consecutive waves targeting specific magnitudes for gross and net change simultaneously in rotating- or split-panel designs. The framework’s focus on both net and gross change estimation allows researchers to make informed decisions specifically about both the sample and survey costs. Part of the challenge in large-scale, nationally representative panel surveys is the planning of efforts to sustain a sample that is representative of the population [12]. The efforts to sustain a representative panel sample are an important part of the success of the panel surveys, and this framework provides a foundation to plan the efforts.

Despite the increasing use of panel surveys and studies employing implicit rotating sample designs and acknowledging their ability to measure change within individuals and over time across all individuals, these survey designs often lack simultaneous power calculations for both aspects [20]. The *nchange* R package aims to address this gap by providing practitioners with the tools to incorporate power calculations in designing such studies. With this package, researchers can better assess the statistical power of their studies for varying levels of sample sizes, and give informed decisions related to survey costs.

## Supporting information

S1 Appendix (DOCX)

## References

[1] Curtin R. (1982), “Indicators of Consumer Behavior: The University of Michigan Surveys of Consumers,” Public Opinion Quarterly, 46(3), 340–352.

[2] Kish L. (1987), Statistical Design for Research. New York: John Wiley & Sons, Inc.

[3] Binder D. A., and Hidiroglou M. A. (1988), “Sampling in Time,” Handbook of Statistics, 6(1), 187–211.

[4] Kasprzyk D., Duncan G., Kalton G., and Singh M. P. (1989), Panel Surveys. New York: J. W. Wiley and Sons, Inc.

[5] Kalton G., and Citro C. F. (1993), “Panel Surveys: Adding the Fourth Dimension,” Survey Methodology, 19, 205–215.

[6] Lynn P. (Ed.). (2009), Methodology of Longitudinal Surveys. John Wiley & Sons.

[7] Duncan G. J., & Kalton G. (1987), “Issues of design and analysis of surveys across time,” International Statistical Review/Revue Internationale de Statistique, 97–117.

[8] Kish L. (1965), Survey Sampling. New York: John Wiley & Sons, Inc.

[9] Johnson, K., Pence, K., and Vine, D. J. (2014), “FEDS Working Paper No. 2014–82,” 10.2139/ssrn.2520172

[10] Tourangeau, R. (2004), “Recurring Surveys: Issues and Opportunities. A Report to the National Science Foundation Based on a Workshop Held on March 28–29, 2003.” https://www.nsf.gov/sbe/ses/mms/nsf04_211a.pdf

[11] Hill, M. S. (1981), “Some Dynamic Aspects of Poverty,” Chapter 3 in Five Thousand American Families: Patterns of Economic Progress, eds. M. S. Hill, D. H. Hill, and J. N. Morgan, Volume 9, University of Michigan, Institute for Social Research, Ann Arbor.

[12] Duncan G. J., Juster F. T., and Morgan J. N. (1987), “The Role of Panel Studies in Research on Economic Behavior,” Transportation Research Part A: General, 21(4–5), 249–263.

[13] Grieger L., Danziger S., and Schoeni R. (2009), “Accurately Measuring the Trend in Poverty in the United States Using the Panel Study of Income Dynamics,” Journal of Economic and Social Measurement, 34. doi: 10.3233/JEM-2009-0313

[14] Census Bureau (2021), “Figure 8. Number in Poverty and Poverty Rate: 1959 to 2020.” https://www.census.gov/content/dam/Census/library/visualizations/2021/demo/p60-273/Figure8.pdf

[15] Census Bureau (2023), “Historical Poverty Tables: People and Families– 1959 to 2021.” https://www.census.gov/data/tables/time-series/demo/income-poverty/historical-poverty-people.html

[16] Ahn C., Heo M., and Zhang S. (2014), Sample Size Calculations for Clustered and Longitudinal Outcomes in Clinical Research. CRC Press.

[17] Valliant R., Dever J., and Kreuter F. (2018), Practical Tools for Designing and Weighting Survey Samples. Springer.

[18] Valliant, R., and Dever, J. A. (2018), Survey Weights: A Step-by-Step Guide to Calculation (p. 183). College Station, TX: Stata Press.

[19] Elkasabi, M. (2023), “nchange: Executes Sample Size Calculations for Longitudinal Surveys” [R package]. https://github.com/mahmoudelkasabi/nchange 10.1371/journal.pone.0291449 PMC10513311 37733701

[20] Lugtig, P. (2021), “What Panel Surveys and Smartphone-App Studies can Learn from Each Other.” Presentation at the 9th Conference of the European Survey Research Association, Online, July 9. https://www.europeansurveyresearch.org/conf2021/uploads/218/623/15/esra_2021_smartphones.pdf

