# Supplementary material for: A framework for sample size calculations in longitudinal surveys to measure net and gross changes
Source: PLoS One. 2023 Sep 21;18(9):e0291449. doi: 10.1371/journal.pone.0291449 (PMC10513311; doi:10.1371/journal.pone.0291449)
Supplement: S1 Appendix — (DOCX) [file pone.0291449.s001.docx]

**Appendix A**

Proof of Equation (4)

When the study variable is a binary outcome with 0/1 values, the means reduce to proportions as below:

$$P_{t}=\frac{1}{n_{t}}\sum_{i\in s_{t}} y_{i}$$

$$P_{t+1}=\frac{1}{n_{t+1}}\sum_{i\in s_{t+1}} y_{i}$$

And element variance and covariance can be written as:

$$\sigma_{x}^{2}=P_{t}\left( 1-P_{t} \right)$$

$$\sigma_{y}^{2}=P_{t+1}\left( 1-P_{t+1} \right)$$

$${\sigma_{xy}=P}_{XY}-P_{t}P_{t+1}$$

Therefore, equation (3) can be re-written as:

$n_{t}=\frac{P_{t}\left( 1-P_{t} \right)+rP_{t+1}\left( 1-P_{t+1} \right)-2\gamma r\left( P_{XY}-P_{t}P_{t+1} \right)}{\delta^{2}}\left( Z_{1-\alpha/2}-Z_{\beta} \right)^{2}$

Proof of Equation (8)

From Equation (3),

$$n_{t}=\frac{1}{\delta^{2}}\left[ \sigma_{x}^{2}+{\frac{n_{t}}{n_{t+1}}\sigma}_{y}^{2}-2\frac{n_{11}}{n_{t+1}}\rho\sigma_{x}\sigma_{y} \right]\left( Z_{1-\alpha/2}-Z_{\beta} \right)^{2}$$

$$\frac{n_{t}\delta^{2}}{\left( Z_{1-\alpha/2}-Z_{\beta} \right)^{2}}=\sigma_{x}^{2}+{\frac{n_{t}}{n_{t+1}}\sigma}_{y}^{2}-2\frac{n_{11}}{n_{t+1}}\rho\sigma_{x}\sigma_{y}$$

$$\frac{n_{t}\delta^{2}}{\left( Z_{1-\alpha/2}-Z_{\beta} \right)^{2}}-\sigma_{x}^{2}=\frac{n_{t}\sigma_{y}^{2}-2n_{11}\rho\sigma_{x}\sigma_{y}}{n_{t+1}}$$

$$n_{t+1}=\frac{n_{t}\sigma_{y}^{2}-2n_{11}\rho\sigma_{x}\sigma_{y}}{\frac{n_{t}\delta^{2}}{\left( Z_{1-\alpha/2}-Z_{\beta} \right)^{2}}-\sigma_{x}^{2}}$$

$$\therefore n_{t+1}=\frac{\left[ {n_{t}\sigma}_{y}^{2}-2n_{11}\rho\sigma_{x}\sigma_{y} \right]\left( Z_{1-\alpha/2}-Z_{\beta} \right)^{2}}{n_{t}\delta^{2}-\sigma_{x}^{2}\left( Z_{1-\alpha/2}-Z_{\beta} \right)^{2}}$$

Proof of Equation (10)

From Equation (4),

$\frac{n_{t}\delta^{2}}{\left( Z_{1-\alpha/2}-Z_{\beta} \right)^{2}}=P_{t}\left( 1-P_{t} \right)+\frac{n_{t}}{n_{t+1}}P_{t+1}\left( 1-P_{t+1} \right)-2\frac{n_{11}}{n_{t+1}}\left( P_{XY}-P_{t}P_{t+1} \right)$

$\frac{n_{t}\delta^{2}}{\left( Z_{1-\alpha/2}-Z_{\beta} \right)^{2}}-P_{t}\left( 1-P_{t} \right)=\frac{n_{t}P_{t+1}\left( 1-P_{t+1} \right)-2n_{11}\left( P_{XY}-P_{t}P_{t+1} \right)}{n_{t+1}}$

$n_{t+1}=\frac{n_{t}P_{t+1}\left( 1-P_{t+1} \right)-2n_{11}\left( P_{XY}-P_{t}P_{t+1} \right)}{\frac{n_{t}\delta^{2}}{\left( Z_{1-\alpha/2}-Z_{\beta} \right)^{2}}-P_{t}\left( 1-P_{t} \right)}$

$${\therefore n}_{t+1}=\frac{\left[ n_{t}P_{t+1}\left( 1-P_{t+1} \right)-2n_{11}\left( P_{XY}-P_{t}P_{t+1} \right) \right]\left( Z_{1-\alpha/2}-Z_{\beta} \right)^{2}}{n_{t}\delta^{2}-P_{t}\left( 1-P_{t} \right)\left( Z_{1-\alpha/2}-Z_{\beta} \right)^{2}}$$

**Appendix B**

Equation (2) can be further simplified as:

$n_{t}= \frac{1}{\delta^{2}}[\sigma_{x}^{2}+ \frac{n_{t}\sigma_{y}^{2}-2n_{11}\sigma_{xy}}{n_{t+1}}] {(Z_{1-\alpha/2}-Z_{\beta})}^{2}$ (B.1)

Then multiple $n_{t+1}$ both sides and denote $A= {(Z_{1-\alpha/2}-Z_{\beta})}^{2}$, we have:

$n_{t}n_{t+1}= \frac{A}{\delta^{2}}\left[ \sigma_{x}^{2}n_{t+1}+ n_{t}\sigma_{y}^{2}-2n_{11}\sigma_{xy} \right]$ (B.2)

After some steps, equation (13) can be written as:

$n_{t+1}= \frac{\frac{A}{\delta^{2}}[n_{t}\sigma_{y}^{2}-2n_{11}\sigma_{xy}]}{n_{t}- \frac{A}{\delta^{2}}\sigma_{x}^{2}}$ = $\frac{\frac{A\sigma_{y}^{2}}{\delta^{2}}n_{t}- \frac{2An_{11}\sigma_{xy}}{\delta^{2}}}{n_{t}- \frac{A}{\delta^{2}}\sigma_{x}^{2}}$ (B.3)

For the simplicity of later calculation, from now on we denote following values:$M= \frac{A\sigma_{y}^{2}}{\delta^{2}}$, $N= - \frac{2An_{11}\sigma_{xy}}{\delta^{2}}$, $K= - \frac{A}{\delta^{2}}\sigma_{x}^{2}$. Therefore, $n_{t+1}= \frac{Mn_{t}+N}{n_{t}+K}$. This is a standard format of fixed-point iteration. To use the iteration, we need one condition:

$$\left| {M \atop1} {N \atop K} \right|\neq0 Condition (a)$$

This is equivalent to $\frac{-A^{2}\sigma_{x}^{2}\sigma_{y}^{2}+2\delta^{2}An_{11}\sigma_{xy}}{\delta^{4}} \neq0$ or $-A^{2}\sigma_{x}^{2}\sigma_{y}^{2}+2\delta^{2}An_{11}\sigma_{xy} \neq0.$

If the *condition (a)* is met, we then can consider problem: $\gamma= \frac{M \gamma+N}{\gamma+K} (B.4)$.

There are three potential cases. The first one is equation (15) has two different roots. The second case it has two identical roots. The last one is it has no root. We will discuss each case in order.

**Case I: Two different roots,** $\boldsymbol{\varphi and \omega}$

In addition to *condition (a), c*$ondition (b) {(K-M)}^{2}+4N>0$ *must be met*.

Then we have:

$$n_{t+1}-\varphi= \frac{Mn_{t}+N}{n_{t}+K}-\varphi= \frac{Mn_{t}+N- \varphi n_{t}- \varphi K}{n_{t}+K} (B.5)$$

Since we assume $\varphi$ is one of the roots, then $\varphi= \frac{M \varphi+N}{\varphi+K}$. That is $N-K\varphi=\varphi^{2}-M\varphi.$Plug this equation into (B.5), we can get:

$$n_{t+1}-\varphi= \frac{Mn_{t}- \varphi n_{t}+\varphi^{2}-M\varphi}{n_{t}+K}= \frac{\left( M- \varphi\right)n_{t}+ \varphi\left( \varphi-M \right)}{n_{t}+K}= \frac{\left( M- \varphi\right)\left( n_{t}- \varphi\right)}{n_{t}+K} (B.6)$$

Following the same argument, we have the same equation for the other root $\omega.$

$$n_{t+1}- \omega= \frac{\left( M- \omega\right)\left( n_{t}- \omega\right)}{n_{t}+K} (B.7)$$

Then divide (B.6) by (B.7) we can get:

$$\frac{n_{t+1}-\varphi}{n_{t+1}- \omega}= \frac{\left( M- \varphi\right)}{\left( M- \omega\right)}\frac{\left( n_{t}- \varphi\right)}{\left( n_{t}- \omega\right)}$$

Then,

$$\frac{n_{t}-\varphi}{n_{t}- \omega}={(\frac{M- \varphi}{M- \omega})}^{n-1}\frac{\left( n_{1}- \varphi\right)}{\left( n_{1}- \omega\right)}$$

After some steps, we have our final formula for this case:

$$n_{t}= \frac{\varphi\left( M- \omega\right)^{n-1}\left( n_{1}- \omega\right)- \omega\left( M- \varphi\right)^{n-1}\left( n_{1}- \varphi\right)}{\left( M- \omega\right)^{n-1}\left( n_{1}- \omega\right)- \left( M- \varphi\right)^{n-1}\left( n_{1}- \varphi\right)}$$

Previously, we assumed ${(K-M)}^{2}+4N>0$. This condition can be further written as $\frac{A^{2}{(\sigma_{x}^{2}+ \sigma_{y}^{2})}^{2}-8A\delta^{2}n_{11}\sigma_{xy}}{\delta^{4}}>0$ or $A^{2}{(\sigma_{x}^{2}+ \sigma_{y}^{2})}^{2}-8A\delta^{2}n_{11}\sigma_{xy}>0$.

Solving $\varphi, \omega$ using Equation (B.4)

$$\varphi= \frac{-K+M+ \sqrt{{(K-M)}^{2}+4N}}{2}$$

$$\omega= \frac{-K+M- \sqrt{{(K-M)}^{2}+4N}}{2}$$

**Case II: Two identical roots** $\boldsymbol{\varphi}$

Now we need $condition \left( c \right): {(K-M)}^{2}+4N=0$. That is $A^{2}{(\sigma_{x}^{2}+ \sigma_{y}^{2})}^{2}-8A\delta^{2}n_{11}\sigma_{xy}=0$.

We can directly solve this root, $\varphi= \frac{M-K}{2}$.

Still use equation (B.6): $n_{t+1}- \varphi= \frac{\left( M- \varphi\right)\left( n_{t}- \varphi\right)}{n_{t}+K}$, when we take inverse of both sides:

$$\frac{1}{n_{t+1}- \varphi}= \frac{n_{t}+K}{\left( M- \varphi\right)\left( n_{t}- \varphi\right)}$$

Now we solve for Q.

$$n_{t}+K=M- \varphi+Q\left( n_{t}- \varphi\right)= \frac{K+M}{2}+Q\left( n_{t}- \varphi\right)$$

$$Q\left( n_{t}- \varphi\right)= n_{t}+K- \frac{K+M}{2}=n_{t}-\varphi$$

$$So Q=1$$

Therefore, $\frac{1}{n_{t+1}- \varphi}= \frac{1}{n_{t}- \varphi}+ \frac{1}{M- \varphi}= \frac{1}{n_{t}- \varphi}+ \frac{2}{M+K}$

Then,

$$\frac{1}{n_{t}- \varphi}= \frac{1}{n_{1}- \varphi}+ \frac{2(n-1)}{M+K}$$

**Case III: No root**

If there is no root for equation (B.4), the fixed-point iteration shown here will not work. We need to find another way to solve the problem.

From the above analysis, it is obvious that all conditions based on the values of $n_{11}, \delta^{2},$ the desired level of significance ($\alpha$) for the net change, and desired power of test $\left( 1-\beta\right)$ for the net change. This implies that the closed form formula exists depends on the question we are trying to solve.

**Appendix C**

Table C.1: Argument Notations of *nchange* Functions

| Functions | Arguments | Notations |
| --- | --- | --- |
| seqmeans | theta | $\theta$ |
|  | rho | $\rho$ |
|  | deff | Design effect |
|  | S2x | $\sigma_{x}^{2}$ |
|  | S2y | $\sigma_{y}^{2}$ |
|  | alt | “one-sided” vs “two-sided” |
|  | del | $\delta$ |
|  | sig.level | $\alpha$ |
|  | power | $\left( 1-\beta\right)$ |
|  | S2o | $\sigma_{o}^{2}$ |
|  | alt.gross | “one-sided” vs “two-sided” |
|  | del.gross | $\Delta$ |
|  | sig.level.gross | $\acute{\alpha}$ |
|  | pow.gross | $\left( 1-\acute{\beta} \right)$ |
| seqprop | theta | $\theta$ |
|  | deff | Design effect |
|  | P1 | $P_{t}$ |
|  | P2 | $P_{t+1}$ |
|  | PXY | $P_{XY}$ |
|  | alt | “one-sided” vs “two-sided” |
|  | sig.level | $\alpha$ |
|  | power | $\left( 1-\beta\right)$ |
|  | P1.gross | $\acute{P}_{t}$ |
|  | P2.gross | $\acute{P}_{t+1}$ |
|  | PXY.gross | $\acute{P}_{XY}$ |
|  | alt.gross | “one-sided” vs “two-sided” |
|  | del.gross | $\Delta$ |
|  | sig.level.gross | $\acute{\alpha}$ |
|  | pow.gross | $\left( 1-\acute{\beta} \right)$ |
